# La pyélonéphrite xanthogranulomateuse

**DOI:** 10.11604/pamj.2016.24.91.9598

**Published:** 2016-05-27

**Authors:** Ali Beddouche, Ousmane Nago Dembele

**Affiliations:** 1 Service d'Urologie A, Hôpital Ibn Sina, CHU Rabat, Maroc

**Keywords:** Pyélonéphrite, xanthogranulomateuse, parenchyme, pseudo-kystes, Pyelonephritis, xanthogranulomatous, parenchyma, pseudocysts

## Image en médecine

Madame LF âgée de 46 ans, sans antécédents particuliers. Elle avait consulté pour des lombalgies gauches. Cliniquement elle était fébrile à 38,8°C, bandelettes urinaires (Nitrites, leucocytes +). L'examen mettait en évidence un contacte lombaire gauche douloureux. Le bilan biologique avait objectivé une hyperleucocytose à 22400/mm^3^, une CRP à 124 mg/l, et l'ECBU a mis en évidence une infection urinaire à Escherichia Coli multi-sensible à l'antibiogramme. Sur le plan radiologique l’échographie abdominale mettait en évidence une importante dilatation pyélocalicielle gauche à contenu finement échogène, avec réduction de l'index corticale. L'uroscanner retrouvait un rein gauche augmenté de taille, siège de multiples cavités liquidiennes intra-parenchymateuses, pseudo-kystiques, hétérogènes et cloisonnées, avec atrophie parenchymateuse majeure, retard excrétoire, et importante infiltration de la graisse péri-rénale, sur une lithiase de l'uretère lombaire de 12mm. L'aspect scannographique nous a permis d’évoquer les diagnostics suivant: une pyélonéphrite xanthogranulomateuse (PXG) diffuse, une pyonéphrose sur rein multi-kystique, une tumeur rénale nécrosée (carcinome a cellules claires…). Une néphrectomie totale élargie a été réalisée, le diagnostic de PXG fut retenu après examen anatomopathologique de la pièce opératoire.

**Figure 1:**
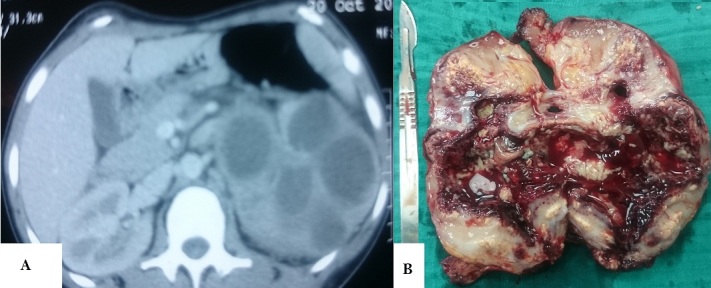
A) TDM: formations pseudo-kystiques rénales gauches; B) pièce de néphrectomie

